# A study on the physical aging characteristics of the older people over 70 years old in China

**DOI:** 10.3389/fpubh.2024.1352894

**Published:** 2024-06-03

**Authors:** Jin-Bo Shen, Yuan-Yuan Ma, Qian Niu

**Affiliations:** ^1^Department of Physical Education, Taiyuan Institute of Technology, Taiyuan, Shanxi, China; ^2^Physical Education Institute of Jishou University, Jishou, Hunan, China

**Keywords:** older people, BMI, blood pressure, muscle strength, flexibility quality

## Abstract

**Aim:**

In China, with the increase of life expectancy and the decrease of fertility rate, the aging problem has become increasingly prominent, and the physical problems of the older people over 70 years are the key and difficult problems.

**Method:**

Based on the interactive logic between the aging problem and the older people health, in the study, a questionnaire survey and a nationwide physical fitness test were carried out on the older people over 70, to divide into different age groups (70–74 years old, 75–79 years old, 80–84 years old, 85 years old and older) and different genders. There were 8,400 valid samples, and 1,050 persons in each group. One-way ANOVA was used to compare the differences among groups of different ages, and a broken line chart was drawn to discuss the aging characteristics of various physical indexes of the older people over 70 in China.

**Result:**

(1) Body morphology: male waist circumference, male waist-to-height ratio and female BMI showed a gradual downward trend with the increase of age; (2) Physiological function: male and female vital capacity showed a decreasing trend with the increase of age, while female pulse pressure showed a gradual upward trend. (3) Physical quality: the indicators of male and female muscle strength, flexibility quality, aerobic endurance and balance showed a downward trend with the increase of age.

**Conclusion:**

Vital capacity, flexibility quality, muscle strength, aerobic endurance, balance ability and so on, decreased significantly with the growth of age. 80 years old is the inflection point of the rapid decline of various indicators. Blood pressure, silent pulse, BMI, waist-to-hip ratio, waist-to-height ratio and other indicators did not change regularly with age. Indicators such as blood pressure, BMI, waist-to-hip ratio and waist-to-height ratio were in the high-risk range of metabolic diseases and cardiovascular and cerebrovascular diseases. The study conducted physical fitness test on the older people over 70 years old in 7 geographical regions of China, which is the first nationwide physical fitness test for the older people, which is an extension and expansion of the national physical fitness monitoring system, and also shows that the test indicators involved in the “Health fitness scale” are simple and feasible. And the study added a series of test data over 70 years old, which is the basis for scientific and reasonable formulation of physical fitness evaluation standards for the older people, and is of great significance for improving the national physical fitness database and grasping the dynamic changes of national physical health status, and providing data support for scientific guidance of physical exercise for the older people.

## 1 Introduction

With the acceleration of the population aging process and the increasingly prominent characteristics of aging, the health problems of the older people have been highly valued by all walks of life in China. The Outline of Healthy China 2030 emphasizes the need to strengthen health guidance and intervention for the older people, and points out that the national constitution should vigorously carry out exercise risk assessment under the health service model of “combination of sports and medicine.” The aging of the older people, especially the older people over 70 years old, itself is the continuation of their life and health, but the extension of life expectancy does not mean the improvement of the quality of life of the older people. Healthy aging is to ensure the increase of life expectancy while maintaining the quality of life. Previous studies have shown that the older people body morphology indicators [like BMI (1), waist circumference (2), waist-to-height ratio (3)] have degenerative changes, and the decline of physiological function and physical quality is the performance of normal biological characteristics. However, the rapid decline of physiological function indicator [like the vital capacity (4)] and physical quality indicators [like strength (5), flexibility (6), balance (7)] will inevitably affect the daily life and behavioral ability of the older people, and may also lead to the difficulty in maintaining psychological and social communication skills, thus affecting the quality of life of the whole family. In this study, data were obtained through physical fitness testing of Chinese older people over 70 years old, and aging characteristics of various physical indexes of older people over 70 years old were analyzed, which is of great significance to grasp the dynamic changes of physical health status of older people.

Surveys have shown that the older people with regular physical exercise habits have a relatively good healthy quality of life, which has a direct or indirect positive effect on physical health. Physical exercise can promote the blood circulation of the whole body, enhance the metabolism of cells and tissues, and is conducive to the supply of oxygen and nutrients to various systems of the body (8). Regular physical exercise mainly improves physical fitness by increasing energy consumption, reducing fat accumulation, controlling body composition, maintaining muscle strength and muscle endurance, cardiopulmonary endurance and flexibility of the older people at a certain level or slowing down the rate of decline (9). The health benefits of physical exercise are mainly reflected by a higher physical fitness level. Through appropriate physical exercise, the human body can be promoted or maintained at a certain physical fitness level, and a certain physical fitness level corresponds to a certain health status (10). Regular physical exercise habits and reasonable arrangement of physical exercise elements can improve the health level, and also provide a reference for the formulation of fitness exercise programs for the older people. Different physical exercise behaviors have different health benefits for the older people. In 2008, the Physical Activity Guidelines for Americans (11) formulated corresponding physical activity guidelines for the older people over 65 years old. At present, China still lacks the physical evaluation standards of the older people over 70 years old, and the physical test data of the older population over 70 years old are lack of scientific guidance for physical activity of the older people.

Therefore, this study obtained data through the nationwide physical fitness test of the older people over 70 years old, and analyzed the aging characteristics of various physical fitness indicators of the older people over 70 years old in China, which is of great significance to grasp the dynamic changes of national physical health status, and can also provide data support for scientific guidance of physical exercise for the older people.

## 2 Test objects and study methods

### 2.1 Test objects

Using multi-stage stratified random cluster sampling method, a physical fitness test was conducted inthe older people over 70 years old from Hainan, Guangdong, Sichuan, Shanxi, Shaanxi, Beijing, Liaoning, Jilin and Anhui provinces. A total of 13,097 people participated in the test, and 9, 145 people completed the test. The criteria of Chinese national physical fitness test and the health and physical fitness test for older people in US is based on the age of 5 years, so does the research. The specific grouping was 70–74 years old, 75–79 years old, 80–84 years old, and over 85 years old, 1,050 people were selected from each gender and age group, and a total of 8,400 people were selected as the statistical sample.

### 2.2 Study methods

#### 2.2.1 Questionnaire survey

A face-to-face questionnaire survey was conducted among the older people over 70 years old. The basic information questionnaire included age, gender, long-term residence, education level, common diseases, exercise history, history of smoking and drinking, history of falls and other social demographic characteristics. The quality-of-life questionnaire (SF-12) was used to investigate the general behavior and cognitive ability of the older people, and the test subjects were preliminarily screened.

#### 2.2.2 Field test

The Healthy Physical Fitness Scale developed by Wang Hongyu (8) for the older people over 70 years old was selected. The index system in the scale was close to the international index system of physical fitness testing for the older people over 70 years old, especially the test indexes designed for Asian race in Japan and Taiwan of China. The scale has good reliability and validity. By the reliability test, 20 people were selected from the 100 samples participating in the test, the Scale reliability coefficient is the two measurements is between 0.767 and 0.954, it shows the measurement index has high measurement stability. By the validity test, the expert score of all secondary indicators is between 7.63 and 8.67, and the standard deviation is between 0.98 and 1.24, indicating that the index system of the scale has good content validity. By Bartlett’s sphericity test, KMO = 0.719 (*p* < 0.01), indicating the existence of common factors among the indicators, suitable for exploratory factor analysis. After orthogonal rotation, four factor components were extracted in combination with the eigenvalue greater than 1, and the eigenvalues were 5.113, 3.917, 2.435, 2.128, and the explanatory variables were 23.242, 17.807, 9.249, 7.856 and 7.759%. The results showed that the health fitness Measurement Table has good structural validity. The table contains three aspects: body shape, physiological function and physical fitness, which can meet the characteristics of testing in indoor or outdoor environment (the required longitudinal space is only 2.44 m). The test items can be used as daily exercise methods for the older people, and the test results can objectively reflect the physical condition of the older people over 70 years old. According to the principle of index screening and index testing, 3 first-level indicators and 16 s-level indicators were finally formed. According to the content of physical test, it is classified as Table 1. Refer to Wang Hongyu’s article “Construction and Application of the evaluation Index System for healthy physical Fitness of the Oldest Old in China” (12). We have recruited the older people over 70 years old under inclusion criteria and exclusion criteria. Inclusion criteria means that participants were required to sign an informed consent, participated the questionnaire survey and physical fitness test are voluntary or agreed by their family members. The test subjects are not limited by chronic diseases, they can move without obstacles independently or with the help of auxiliary tools, have certain cognitive ability, can independently complete the indicators of physical fitness test, and can answer the questions of the test staff. Exclusion criteria means that people under 70 years old, have cognitive impairment, cannot answer questions effectively, have a physical disability which prevents them from completing any of the test criteria, inability to complete any of the test items, the body is obviously weak.

**Table 1:** The index system of physical fitness test for the older people over 70 years old.

First level indicators	Second level indicators	
Body morphology index	BMI, Waist circumference, Waist-to-hip ratio, Waist-to-height ratio	
Physiological function index	Systolic pressure, diastolic pressure, Pulse pressure, Quiet pulse, vital capacity	
Physical quality index	Flexibility	Hands clasped back, Seat bent forward
	Strength and speed	Grip strength, 30 s or 5 times upper arm dumbbell bends, 30 s or 5 times sit-station trials
		2 min standing still
	Endurance	2.44 m chair stand up and walk around the object
	Balance	

### 2.3 Data processing

In this study, SPSS21.0 software was used to collate and input test data, and measurement data were presented as ^−^*x* ± *s*. One-way analysis of variance was used to compare the differences among different age groups with the same sex, with a significant level of 0.05.

## 3 Results

### 3.1 The results were compared among different age groups

In this study, the body shape test, physiological function test and physical fitness test were carried out on the older people aged over 70 in different age groups. The test results are shown in Tables 2–4. In order to ensure the safety principle of the older people, the two indicators of upper arm dumbbell bending and sitting test in different age groups have the same test content, but the units are different, so the unit conversion is carried out when comparing different age groups, and the unified unit is s/time.

**Table 2:** The results of body morphology test for people over 70 years old (*x̄* ± *s*, *n* = 1,050).

Indicators		70 ~ 74 years old	75 ~ 79 years old	80 ~ 84 years old	Over 85 years old
BMI	Male	24.5 ± 2.4	24.0 ± 3.3^▲^	24.0 ± 2.8^▲^	24. 1 ± 2.9^▲^
	Female	24.0 ± 2.9	23.3 ± 2.7^▲^	23. 1 ± 2.9^▲^	22.6 ± 3.0^▲●◆^
Waist line(m)	Male	0.898 ± 0.076	0.890 ± 0.092^▲^	0.878 ± 0.094^▲●^	0.861 ± 0.088^▲●◆^
	Female	0.832 ± 0.077	0.835 ± 0.068	0.830 ± 0.094	0.821 ± 0.094^▲●◆^
Waist-to-hip ratio	Male	0.94 ± 0.06	0.94 ± 0.06	0.94 ± 0.06^▲●^	0.94 ± 0.05
	Female	0.89 ± 0.05	0.90 ± 0.06	0.89 ± 0.06	0.90 ± 0.06^▲●◆^
Waist-to-height ratio	Male	0.54 ± 0.04	0.54 ± 0.06	0.53 ± 0.05^▲●^	0.53 ± 0.05^▲●◆^
	Female	0.54 ± 0.05	0.54 ± 0.06	0.54 ± 0.06	0.53 ± 0.06^●^

Compared with 70 ~ 74 years old group, ^▲^*p* < 0.05; compared with 75 ~ 79 years old group, ^●^*p* < 0.05; compared with 80 ~ 84 years old group, ^◆^*p* < 0.05.

**Table 3:** The results of physiological function test for people over 70 years old (*x̄* ± *s*, *n* = 1,050).

Indicators		70 ~ 74 years old	75 ~ 79 years old	80 ~ 84 years old	Over 85 years old
Systolic pressure (mmHg)	Male	137.0 ± 13.2	145.0 ± 15.5^▲^	143.3±11.6^▲●^	143.6 ± 12.1^▲●^
	Female	141.3±14.8	139.6±11.5^▲^	142.0±12.7^●^	142.8 ± 12.56^▲●^
Diastolic pressure (mmHg)	Male	79.8 ± 10.2	83.8 ± 9.6^▲^	81.0 ± 8.6^▲●^	81.3 ± 9.9^▲●^
	Female	83.0 ± 10.4	83.3 ± 8.6	82.9 ± 8.4	83.9 ± 8.7^▲◆^
Pulse pressure (mmHg)	Male	57.2 ± 10.7	61.2 ± 11.6^▲^	62.3 ± 11.7^▲●^	62.3 ± 11.4^▲●^
	Female	58.3 ± 11.4	56.2 ± 10.5^▲^	59.0 ± 11.9^●^	58.8 ± 11.3^●^
Quiet pulse(times/min)	Male	75.4 ± 8.6	76.3 ± 7.8^▲^	75.867.8	75.6 ± 7.3^●^
	Female	77.1 ± 7.7	78.1 ± 7.9^▲^	76.7 ± 7.1^●^	77.8 ± 7.6^▲◆^
Vital capacity(ml)	Male	2,531 ± 5,53	2,364 ± 421^▲^	2081 ± 412^▲●^	1839 ± 396^▲●◆^
	Female	1887 ± 4,09	1,651 ± 385^▲^	1,491 ± 356^▲●^	1,161 ± 282^▲●◆^

Compared with 70 ~ 74 years old group, ^▲^*p* < 0.05; compared with 75 ~ 79 years old group, ^●^*p* < 0.05; compared with 80 ~ 84 years old group, ^◆^*p* < 0.05.

**Table 4:** The results of physical quality test for people over 70 years old (*x̄* ± *s*, *n* = 1,050).

Indicators		70-74 years old	75 ~ 79 years old	80 ~ 84 years old	Over 85 years old
Hands clasped back (cm)	Male	−10.4 ± 8.2	−13.5 ± 9.3^▲^	−18.8 ± 8.8^▲●^	−22.7 ± 8.6^▲●◆^
	Female	−8.9 ± 8.1	−14.0 ± 6.9^▲^	−17.4 ± 8.2^▲●^	−20.4 ± 7.9^▲●◆^
Seat bend forward (cm)	Male	−6.7 ± 9.4	−9.2 ± 9.7^▲^	−15.9 ± 9.9^▲●^	−19.6 ± 9.6^▲●◆^
	Female	−4. 1 ± 8.2	−10.2 ± 8.9 ^▲^	−13.3 ± 8.3^▲●^	−15.5 ± 8.7 ^▲●◆^
Grip strength(kg)	Male	31.8 ± 4.8	28.3 ± 5.0 ^▲^	24.7 ± 4.3^▲●^	22.5 ± 3.6^▲●◆^
	Female	20.4 ± 4.0	18.8 ± 3.0^▲^	16.7 ± 2.6^▲●^	13.0 ± 2.3^▲●◆^
Upper arm dumbbell bends(s/time)	Male	1.67 ± 0.46	1.93 ± 0.61^▲^	4. 16 ± 1.16^▲●^	4.77 ± 1.16^▲●◆^
	Female	1.87 ± 0.55	2.12 ± 0.60^▲^	3.97 ± 1.00^▲●^	4.22 ± 1.04^▲●◆^
Sit-station trails (s/time)	Male	1.93 ± 0.49	2.30 ± 0.67^▲^	4.42 ± 1.21^▲●^	4.92 ± 1.01^▲●◆^
	Female	2.22 ± 0.62	2.26 ± 0.58	4.24 ± 0.85^▲●^	4.60 ± 1.02^▲●◆^
2 min standing still (times)	Male	82.5 ± 13.2	78. 1 ± 12.4 ^▲^	71.2 ± 12.5^▲●^	62.5 ± 10. ^▲●◆^
	Female	78.8 ± 12.7	72.3 ± 11.7^▲^	66.8 ± 11.7^▲●^	59.8 ± 10.9^▲●◆^
2.44 m chair stand up and walk around the object(s)	Male	7.84 ± 1.78	8.90 ± 1.75^▲^	10.40 ± 2.31^▲●^	12.64 ± 2.49^▲●◆^
	Female	8.35 ± 2.03	9.97 ± 2.04^▲^	11.65 ± 2.37^▲●^	13.50 ± 2.41^▲●◆^

Compared with 70 ~ 74 years old group, ^▲^*p* < 0.05; compared with 75 ~ 79 years old group, ^●^*p* < 0.05; compared with 80 ~ 84 years old group, ^◆^*p* < 0.05.

### 3.2 The change trend of physical aging in older people over 70 years old

The index data of each group of physical fitness test samples were used to draw a line chart to analyze the trend of physical aging of the older people over 70 years old.

#### 3.2.1 Body morphology

##### 3.2.1.1 BMI

**Figure 1:**
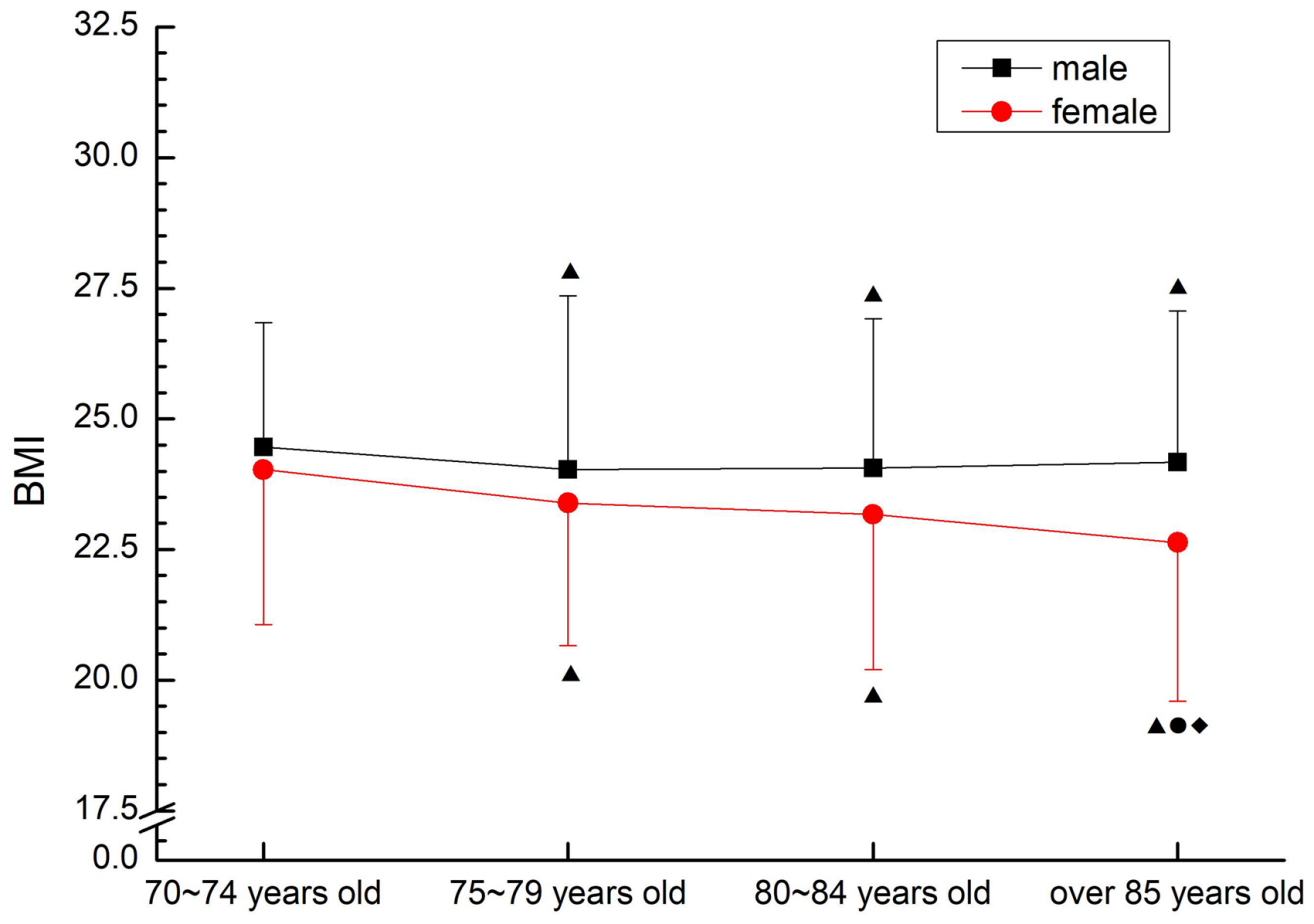
The aging trend of BMI.

In males, it decreased first and then stabilized, and the 70–74 years old group was significantly higher than that in other groups (*p* < 0.05). Females showed a gradual downward trend, and the 70–74 years old group was significantly higher than other groups, and the 85 years old group was significantly lower than other groups (*p* < 0.05) (see Figure 1).

##### 3.2.1.2 Waist circumference

**Figure 2:**
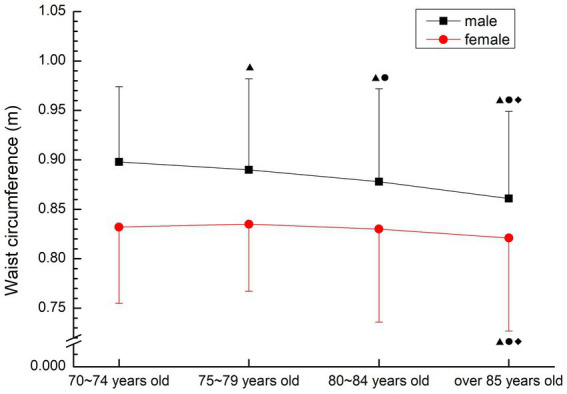
The aging trend of waist circumference.

In males, there was a downward trend, and there were significant differences among all groups (*p* < 0.05). The mean waist circumference of female aged 75–79 years was the largest, and showed a trend of first increasing and then decreasing, while the mean waist circumference of female aged over 85 years was significantly lower than that of other groups(*p* < 0.05) (see Figure 2).

##### 3.2.1.3 Waist-to-hip ratio

**Figure 3:**
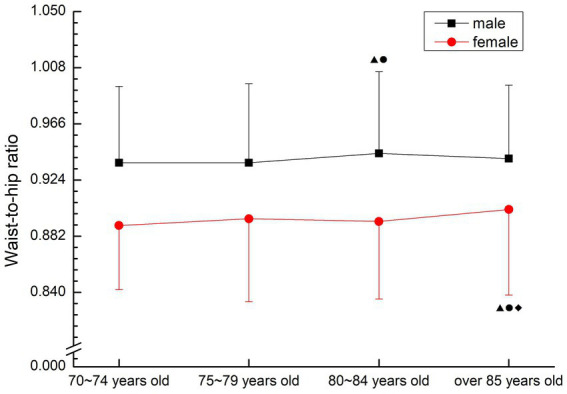
The aging trend of waist-to-hip ratio.

There was no obvious aging trend for males and females. The male 80–84 years old group was significantly higher than the previous two age groups (*p* < 0.05), and the male over 85 years old group was significantly higher than other groups (*p* < 0.05). Female 85 years old group was significantly higher than other groups (*p* < 0.05) (see Figure 3).

##### 3.2.1.4 Waist-to-height ratio

**Figure 4:**
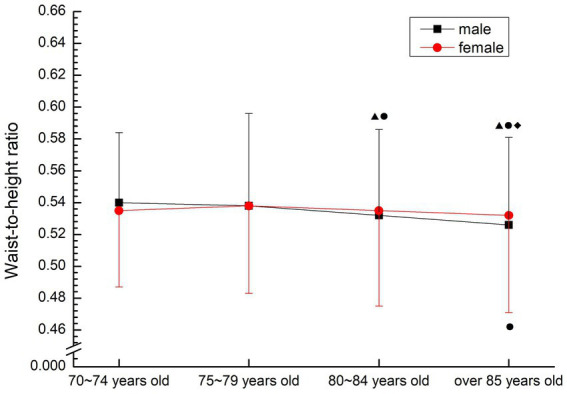
The aging trend of waist-to-height ratio.

The male group showed a gradual downward trend, and the 80–84 years old group was significantly higher than the 70–74 years old group and the 75–79 years old group (*p* < 0.05), and the 85 years old group was significantly higher than other groups (*p* < 0.05). In females, it was increased first and then decreased, but the group over 85 years old was significantly lower than that in 75–79 years old group (*p* < 0.05) (see Figure 4).

#### 3.2.2 Physiological function

##### 3.2.2.1 Systolic pressure

**Figure 5:**
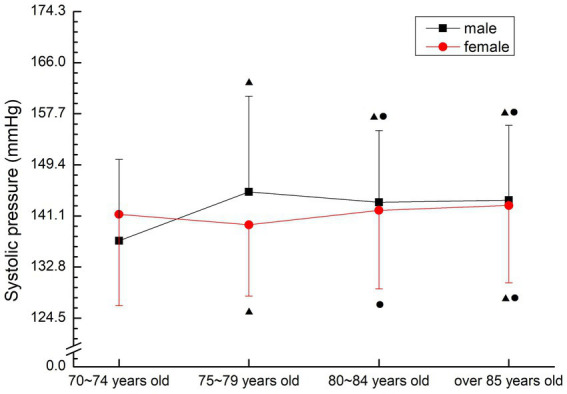
The aging trend of systolic pressure.

There was no regular change between males and females. Male 70–74 years old group was significantly lower than other groups (*p* < 0.05), 80–84 years old group, 85 years old group was significantly lower than 75–79 years old group (*p* < 0.05); The female 75–79 years old group was significantly lower than the 70–74 years old group (*p* < 0.05), the female over 85 years old group was significantly higher than the 70–74 years old group (*p* < 0.05), the female 80–84 years old group and the female over 85 years old group were significantly higher than the 75–79 years old group (*p* < 0.05) (see Figure 5).

##### 3.2.2.2 Diastolic pressure

**Figure 6:**
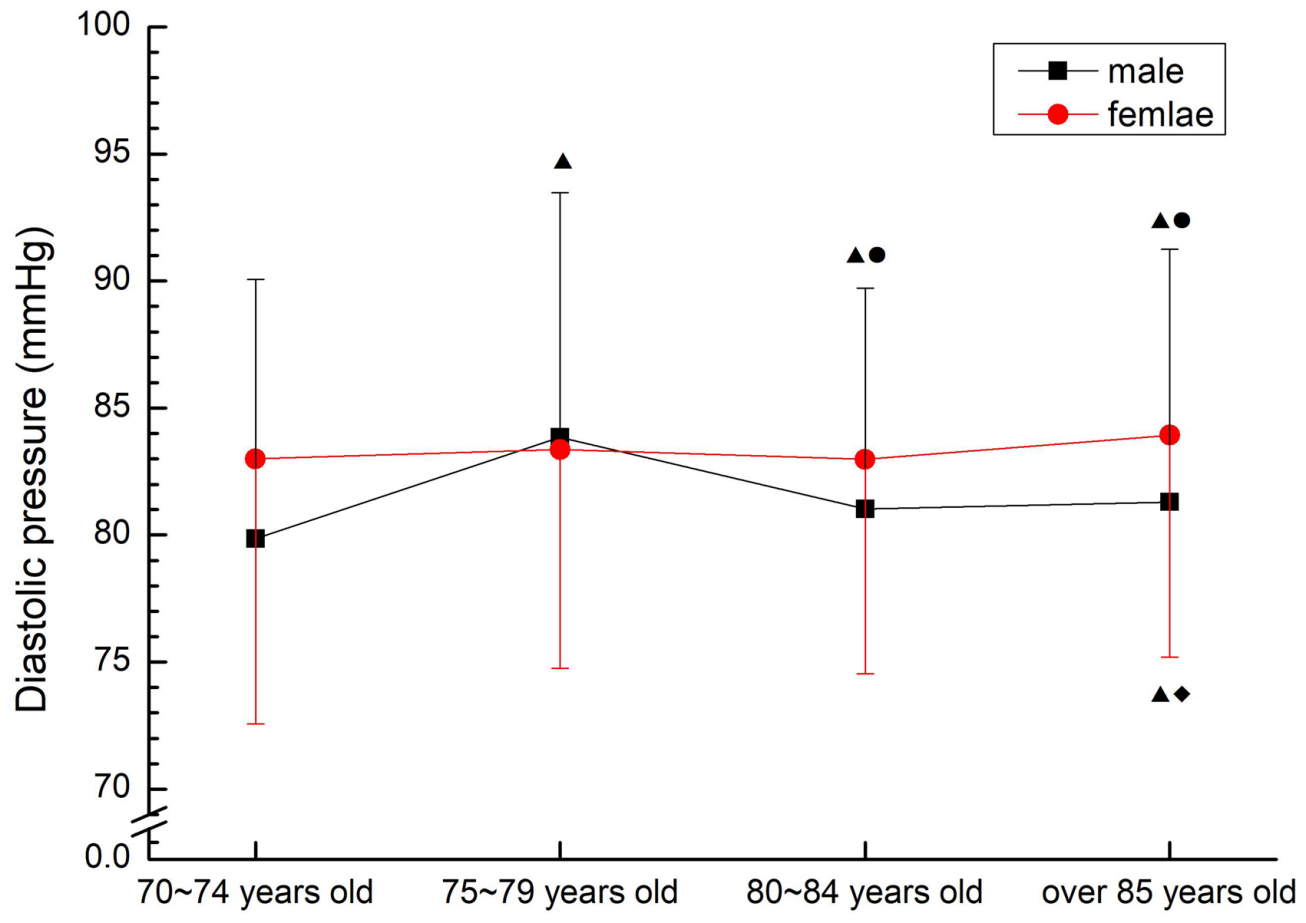
The aging trend of diastolic pressure.

Male 70–74 years old group was significantly lower than other groups (*p* < 0.05), 80–84 years old group and 85 years old group were significantly lower than 75–79 years old group (*p* < 0.05), 80–84 years old group had no significant change compared with 85 years old group (*p* > 0.05); Females were relatively stable, and the group over 85 years old was significantly higher than that in 70–74 years old and 80–84 years old (*p* < 0.05) (see Figure 6).

##### 3.2.2.3 Pulse pressure

**Figure 7:**
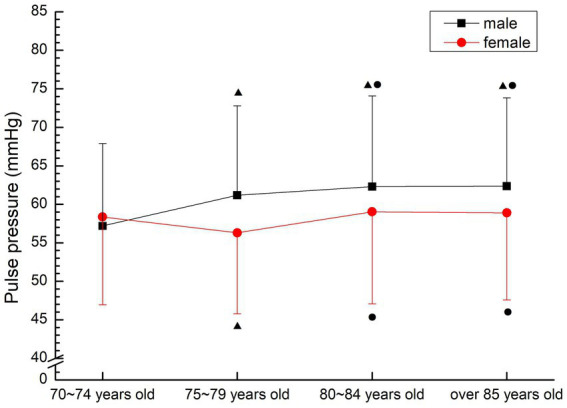
The aging trend of pulse pressure.

In males, it was decreased first and then increased, and the 70–74 years old, 80–84 years old and over 85 years old groups were significantly higher than the 75–79 years old group (*p* < 0.05). Females showed a gradual upward trend, and the 75–79 years old group, the 80–84 years old group and the 85 years old group were significantly higher than the 70–74 years old group (*p* < 0.05), and the 80–84 years old group and the 85 years old group were significantly higher than the 75–79 years old group (*p* < 0.05) (see Figure 7).

##### 3.2.2.4 Quiet pulse

**Figure 8:**
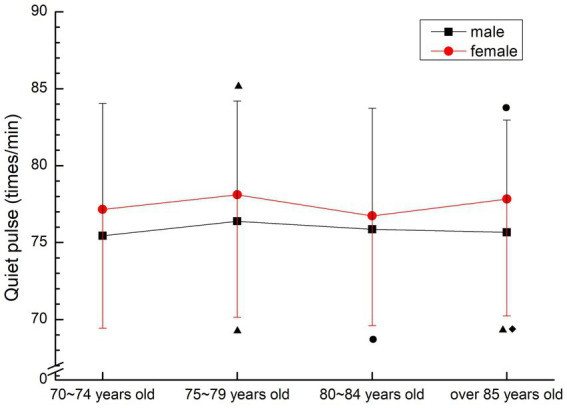
The aging trend of quiet pulse.

There was no change of age between males and females. Male 75–79 years old group was significantly higher than that of 70–74 years old group and over 85 years old group (*p* < 0.05); The female 75–79 years old group was significantly higher than the 70–74 years old group and the 80–84 years old group (*p* < 0.05), and the over 85 years old group was significantly higher than the 70–74 years old group and the 80–84 years old group (*p* < 0.05) (see Figure 8).

##### 3.2.2.5 Vital capacity

**Figure 9:**
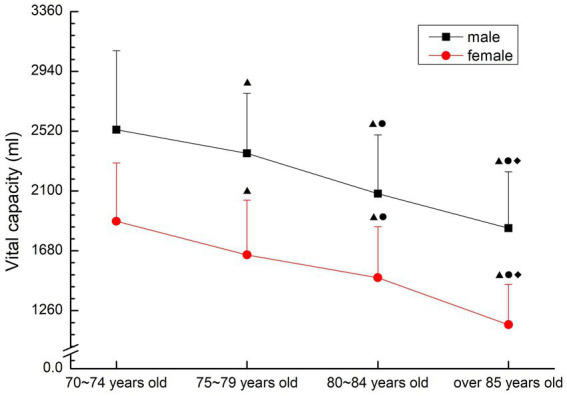
The aging trend of vital capacity.

Both males and females showed a decreasing trend with the increase of age. There were significant differences among all groups (*p* < 0.05) (see Figure 9).

#### 3.2.3 Physical quality

##### 3.2.3.1 Hands clasped back

**Figure 10:**
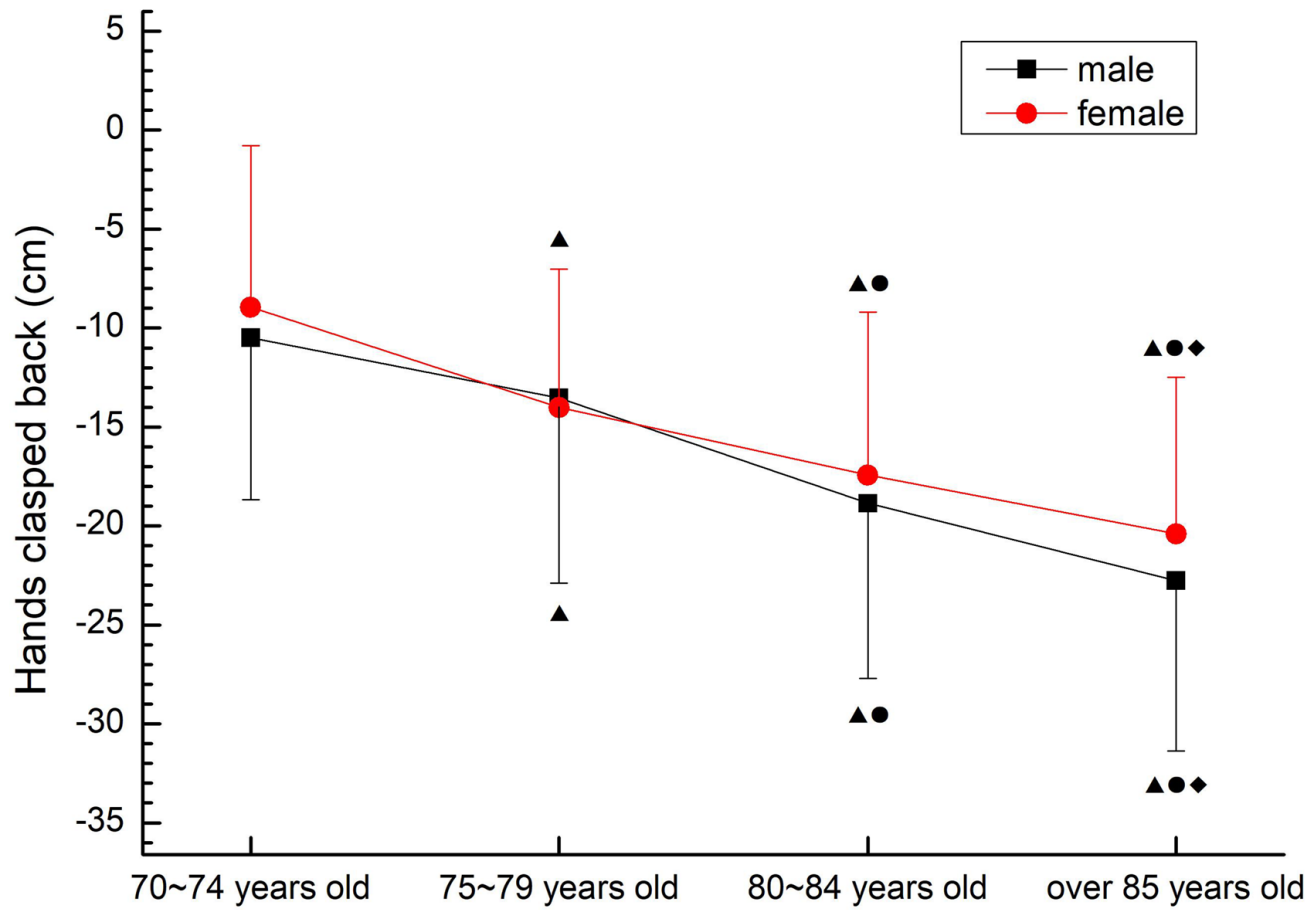
The aging trend of hands clasped back.

The upper limb flexibility of males and females showed a rapid decline trend. There were significant differences among all groups (*p* < 0.05). The decline was fastest in the 75–79 and 80–84 age groups for male, and fastest in the 70–74 and 75–79 age groups for female (see Figure 10).

##### 3.2.3.2 Seat bend forward

**Figure 11:**
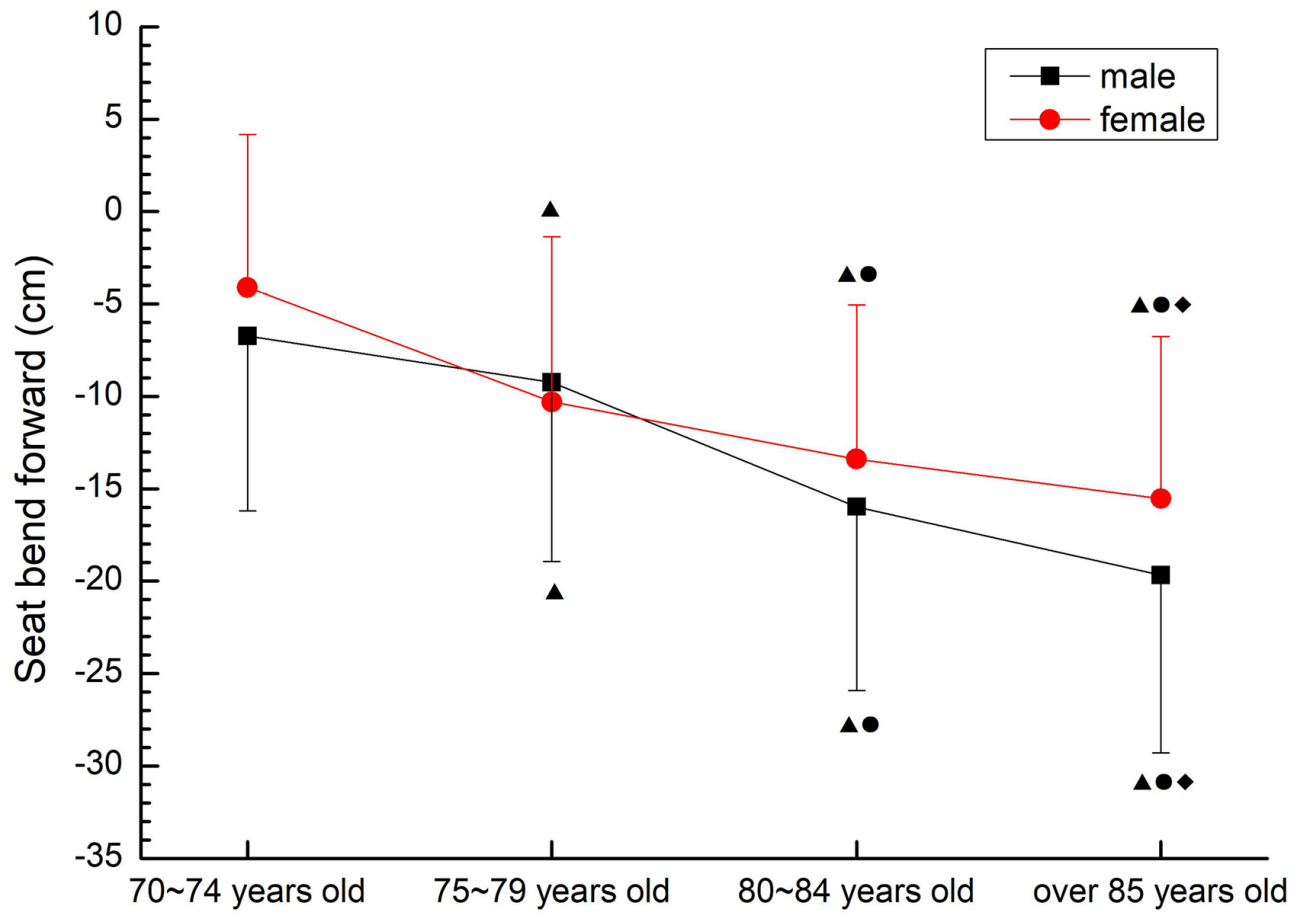
The aging trend of seat bend forward.

The lower limb flexibility of males and females showed a rapid decline trend. There were significant differences among all groups (*p* < 0.05). The decline was fastest in the 75–79 and 80–84 age groups for male, and fastest in the 70–74 and 75–79 age groups for female (See Figure 11).

##### 3.2.3.3 Grip strength

**Figure 12:**
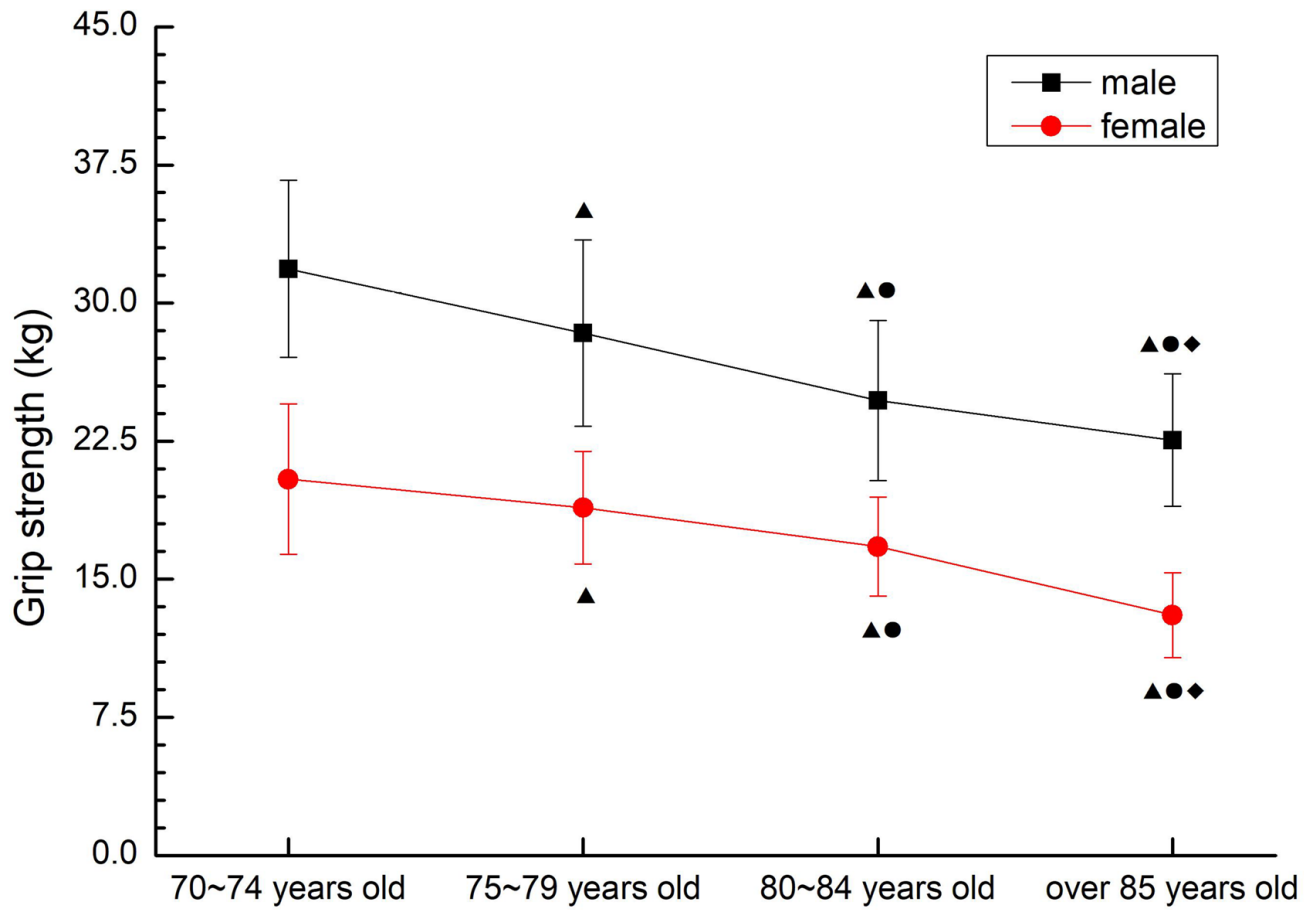
The aging trend of grip strength.

Both males and females showed a gradual downward trend. There were significant differences among all groups (*p* < 0.05) (see Figure 12).

##### 3.2.3.4 Upper arm dumbbell bend

**Figure 13:**
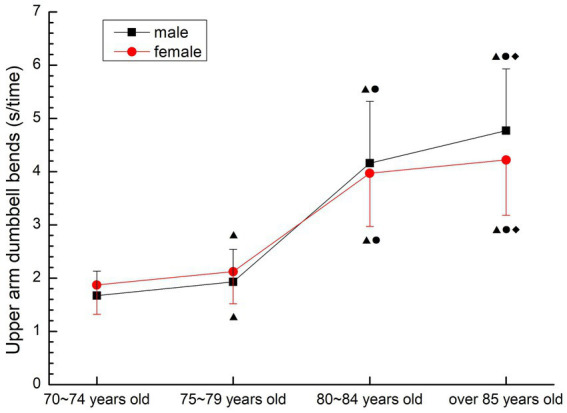
The aging trend of upper arm dumbbell bend.

The change trend of upper limb muscle endurance of males and females was basically the same, and the time required to complete the movement gradually increased, and the change was most obvious in 75–79 years old group and 80–84 years old group. There were significant differences among all groups (*p* < 0.05) (see Figure 13).

##### 3.2.3.5 Sit-station trails

**Figure 14:**
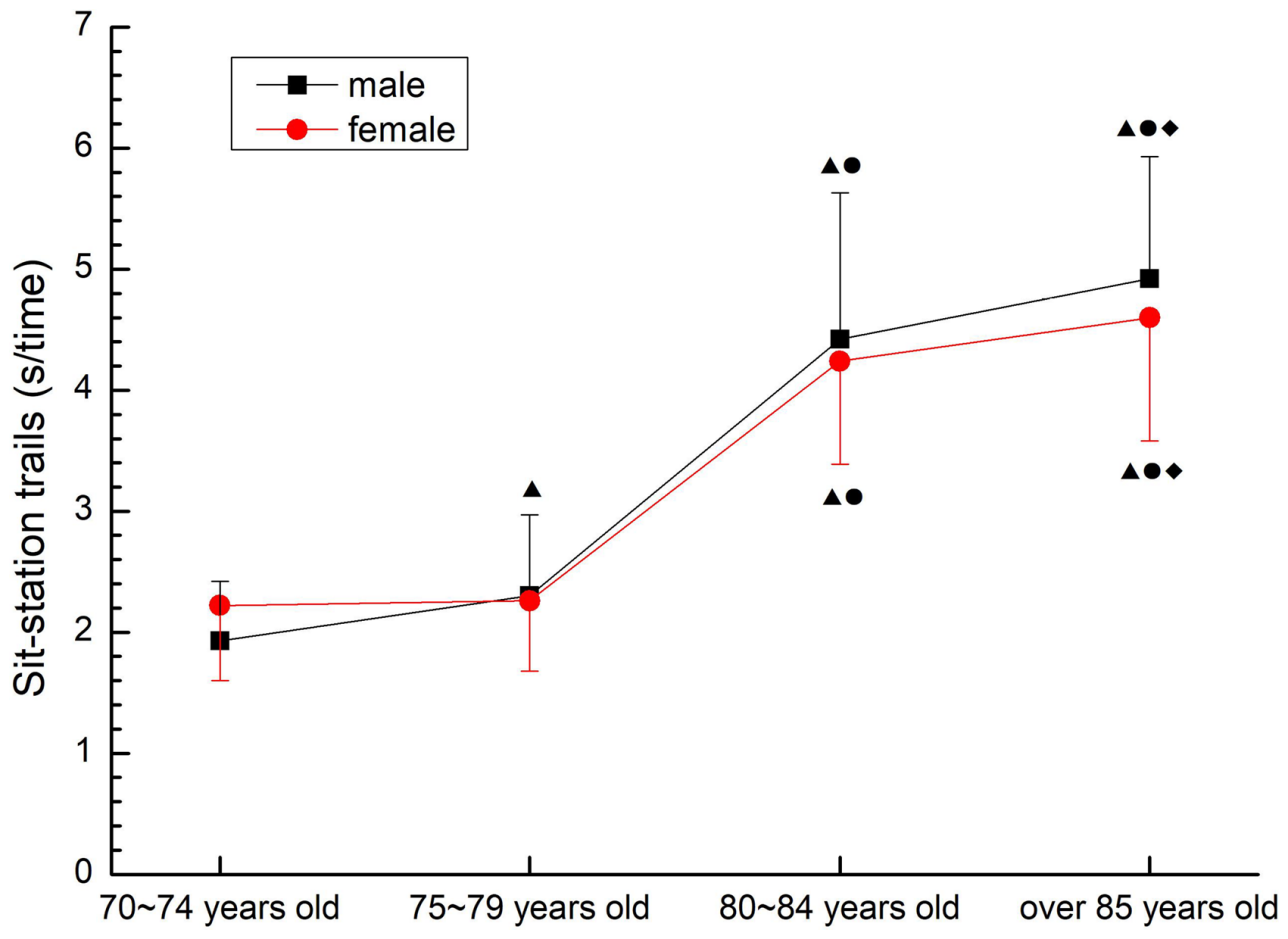
The aging trend of sit-station trails.

The change trend of males and females was basically the same, the time required to complete the movement gradually increased, and the change was most obvious in males and females aged 75–79 and 80 ~ 84. There were significant differences among all groups (p < 0.05) except female 70–74 years old group and 75–79 years old group (*p* > 0.05) (see Figure 14).

##### 3.2.3.6 2 min Standing still

**Figure 15:**
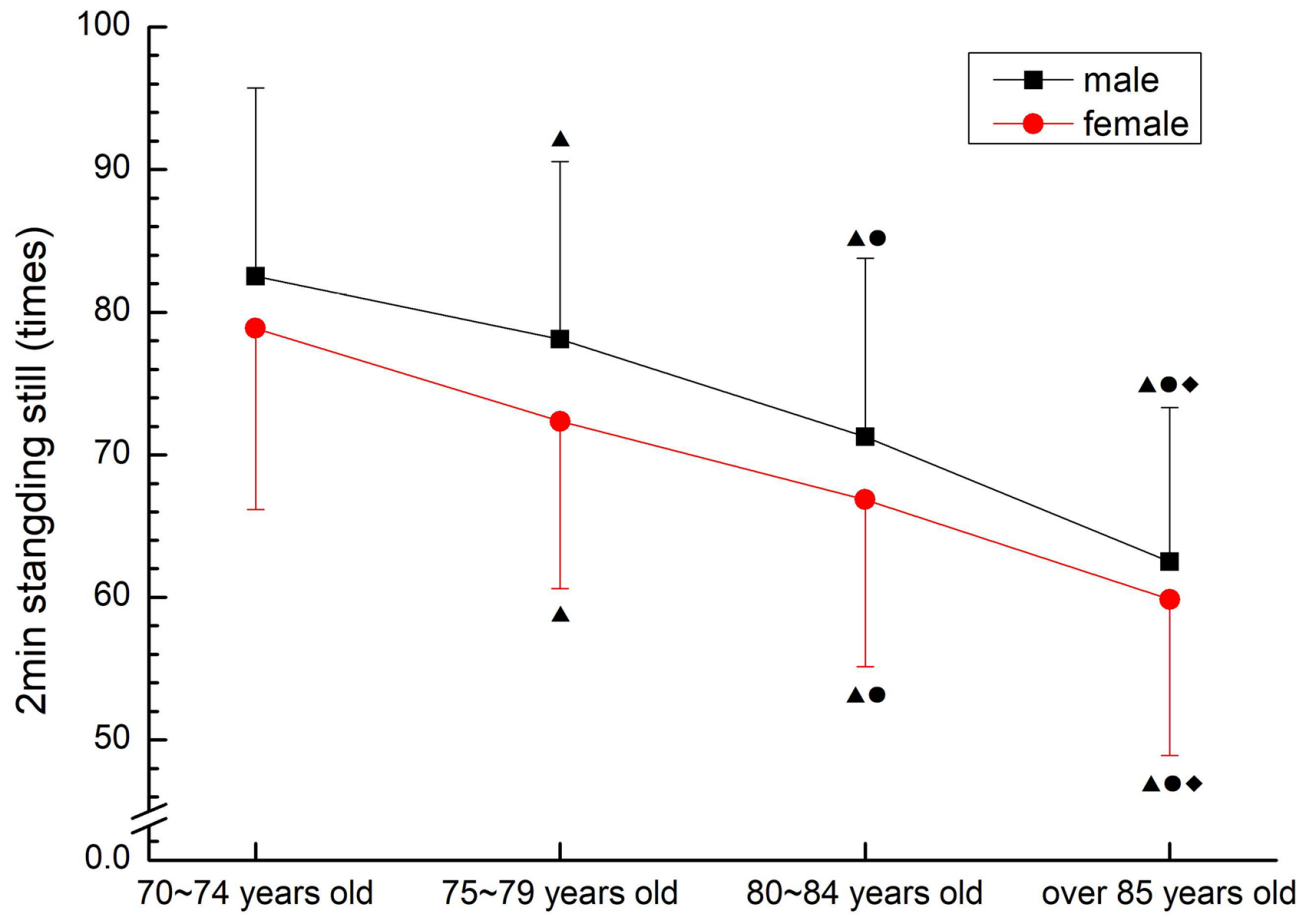
The aging trend of standing still.

Both males and females showed a gradual downward trend. There were significant differences among all groups (*p* < 0.05) (see Figure 15).

##### 3.2.3.7 2.44 m Chair stand up and walk around the object

**Figure 16:**
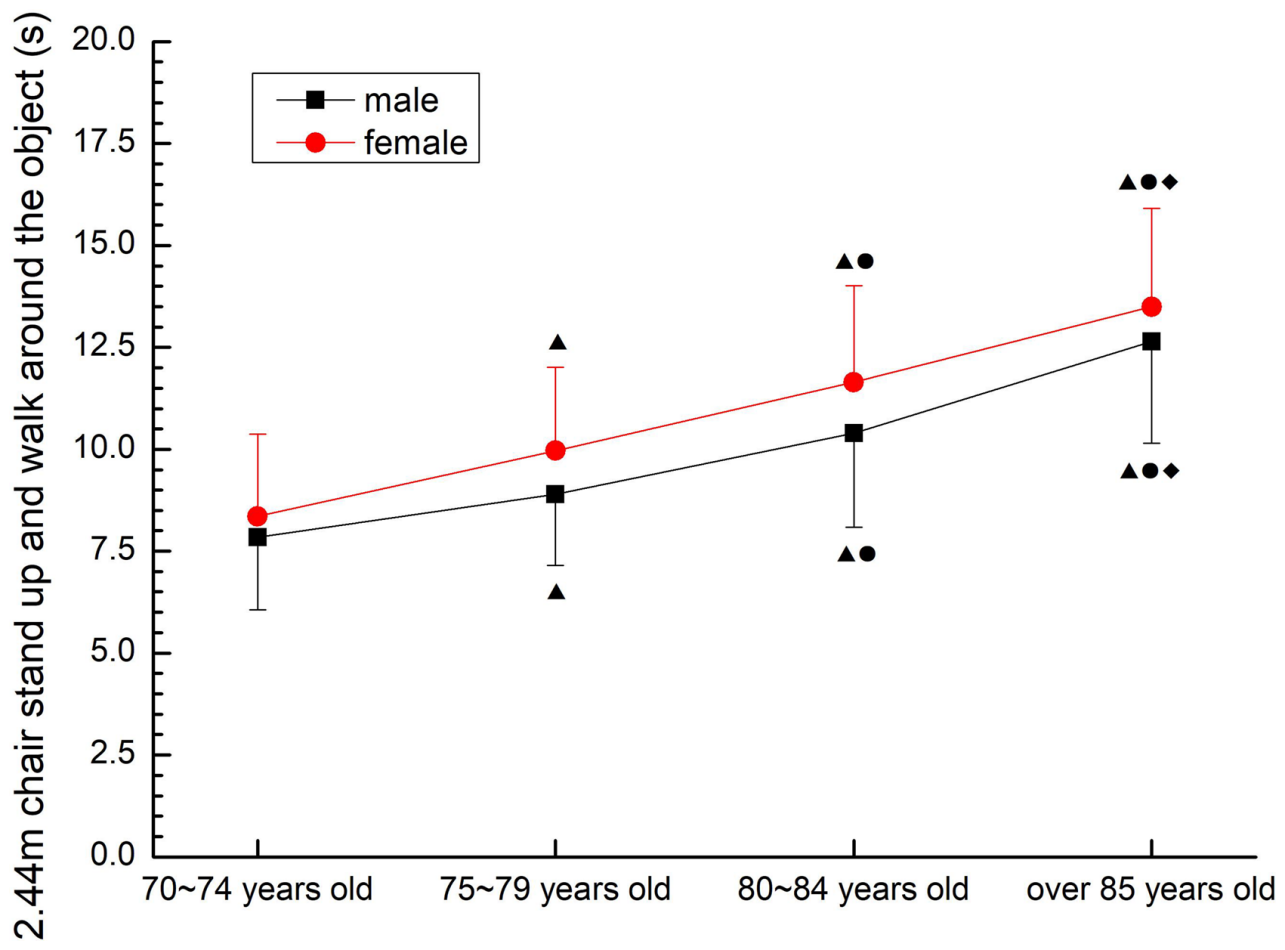
The aging trend of 2.44 m chair stand up and walk around the object.

The time required for both males and females to complete the movements was gradually extended. There were significant differences among all groups (*p* < 0.05) (see Figure 16).

## 4 Analysis and discussion

### 4.1 The analysis of aging characteristics of body morphology in older people over 70 years old

BMI (Body Mass Index) is an important index to evaluate the degree of adult obesity, which is closely related to body fat content. The method of BMI measurement is simple and the other groups practical. According to the health industry standard (WS/T) (13), a BMI of 24 ~ 27.9 is overweight, and a BMI greater than 28 is obese. The results of this study showed that the mean BMI of male older people over 70 years old was at the overweight level, and only decreased between the 70–74 years old group and the 75–79 years old group (*p* < 0.05), and there was no significant change in (*p* > 0.05). Females are at normal levels, with emaciation and malnutrition, and a gradual decline with age. The BMI results of this study are similar to the weight results of the 60-year-old older people in the same period: The proportions of “normal weight,” “underweight,” “overweight” and “obese” are 66, 8.3%, 23.1, and 2.6%, respectively (1), that is, there is no obvious change in BMI of the older people when they grow from 60 years old to 70 years old. As the height of the older people does not change much with age, BMI is closely related to weight. Overweight and obesity are independent risk factors for coronary heart disease and stroke. In this study, the high BMI of males over 70 years old increased the incidence of coronary heart disease and stroke. The relative risk of coronary heart disease, stroke and ischemic stroke increased by 15.4%, 6.1, and 18.8% for each increase in BMI of 2. Once it reaches or exceeds 24, the probability of suffering from high blood pressure, diabetes, coronary heart disease and dyslipidemia and other serious health diseases will increase significantly, suggesting that older people male over 70 years old should strengthen physical activity and physical exercise to reduce or maintain weight.

Waist circumference is a diagnostic indicator for determining metabolic syndrome by the International Diabetes Federation and an important indicator for determining central obesity (2), meanwhile, Hara (14) pointed out that waist-to-hip ratio and waist-to-height ratio were more accurate than BMI in predicting risk factors. Waist-to-hip ratio is a common method to predict the risk of obesity and heart disease. If the ratio is greater than 0.9 for male and 0.8 for female, it can be diagnosed as central obesity, which is three times more accurate than the method of measuring BMI. Large waist size indicates that fat exists in the abdomen, which is a signal of greater risk. Waist-to- height ratio can effectively reflect the accumulation of abdominal fat, especially visceral fat, such as heart, liver, kidney, etc. If waist-to-height ratio is too large, the incidence of hypertension, coronary heart disease, myocardial infarction, stroke, diabetes and other diseases will increase (3). Stratification of disease risk using a specific combination of BMI and waist-to-hip ratio identifies the risk of obesity and serves as an independent predictor of disease risk, predicting the degree of cardiorespiratory fitness within the current BMI and waist circumference range (15). In this study, the waist-to-hip ratio of the older people over 70 years old was far higher than the average level of normal adults, and there was no significant decrease with age (*p* > 0.05). The waist-to-height ratio of normal adults is below 0.5, and the waist-to-height ratio of older people over 70 is slightly higher than that of adults. High waist-to-hip ratio suggests that the older people over 70 years old have a high risk of suffering from metabolic syndrome and other diseases, and are at high risk for many related diseases. Therefore, attention should be paid to posture control and exercise intensity when participating in physical exercise.

The aging trend of various indicators of body shape of the older people over 70 years old is not obvious, which may be due to the fact that physical exercise and diet have more significant effects on body shape than gender and age, and it is difficult to reflect the differences in body shape from the perspective of gender and age.

### 4.2 The analysis of aging characteristics of physiological function in older people over 70 years old

Blood pressure can determine cardiac function and peripheral vascular resistance, and is an important part of diagnosing diseases, observing changes in conditions and judging treatment effects (16). Many scholars believe that the current diagnostic criteria for hypertension is no longer the gold standard, and the evaluation criteria for hypertension of different gender and age groups should have different critical ranges (17). The normal blood pressure range of normal older people gradually increases with age, and the reference range of normal blood pressure increases by 10 mmHg for every 10 years of age. However, since the medical research on hypertension in 1896, the criteria for determining hypertension have been further lowered. In 2003, the new recommended guidelines for hypertension in the United States took the blood pressure value of 115/75 mmHg as the new standard (18). In this study, the average blood pressure was in the hypertensive or critical state of the current hypertension criteria, and did not show regular changes with age. The average pulse pressure was also in the high-risk range of cardiovascular diseases, indicating poor oxygen transport capacity and high risk of hypertension-related diseases. Therefore, the prevention of cardiovascular and cerebrovascular diseases in the older people should be considered.

The quiet pulse of normal older people slowed down significantly, but most of them were still in the normal range (60–100 beats/min). Chen et al. (19) believed that the slow heart rate of the older people was caused by the aging of the sinoatrial node, and other studies believed that the slow heart rate of the older people was caused by the diffusion of myocardial sclerosis and calcification to the cardiac conduction system (20). In this study, the mean quiet pulse was in the normal range of adults, in line with the characteristics of adult quiet pulse, but generally higher than the ideal pulse, indicating that the heart conduction function of the older people over 70 years old did not significantly decline and maintained a relatively stable heart condition, which may be related to the exclusion of subjects suffering from heart disease in the screening scope of this study.

Vital capacity is the most intuitive and objective indicator for detecting lung function (21). In this study, the vital capacity decreased gradually with the increase of age, and the difference among all age groups was significant (*p* < 0.05), indicating that the lung function of the older people over 70 years old showed a gradual decline trend with the change of age. The main reason for the decline in oxygen transport capacity in the older people is the gradual decline in the functions of important organs, including the heart and lungs, and the decrease in the elasticity of the blood vessel wall, which leads to large fluctuations in blood pressure, and the weakening of the elasticity of the alveoli, which leads to the gradual decline in cardiopulmonary function. Studies have shown that jogging can effectively reduce the heart rate, systolic blood pressure, diastolic blood pressure in the older people; at the same time, it can increase the vital capacity and maximum gas volume of the older people (4). Both Taijiquan (22) and walking (23) can effectively improve cardiovascular function and play a role in preventing and treating diseases. Exercise can regulate respiratory function and play a positive role in improving cardiopulmonary function. It is suggested that the older people should actively participate in physical activities to maintain the elasticity of blood vessels and the ability of blood transport. At the same time, appropriate environment and scientific quantitative exercise stimulation are needed to maintain the relative stability of lung function.

### 4.3 The analysis of aging characteristics of physical quality in older people over 70 years old

The physical fitness of the older people over 70 years old decreased significantly with age, and the decline trend of strength, speed, endurance, flexibility, balance and other qualities was basically the same.

#### 4.3.1 Strength, strength endurance quality

As a consistent method to detect muscle strength, grip strength is an important indicator reflecting the muscle strength of the human upper limb, and is highly correlated with muscle strength in other parts of the body, so it is often used as a common indicator in physical testing and epidemiological studies (24). Considering that the older people over 70 years old completed more daily activities of upper limb activities, the upper arm dumbbell bending was added to the completion of the grip strength test to evaluate the upper limb muscle strength endurance. The lower limb muscle strength test was completed by the sitting-up and standing test modified by the “continuous sitting chair” test. This method has been proved to be feasible for the evaluation of lower limb muscle strength in the older people, and the sitting-standing test is considered to be a simple experiment and exercise method with reliable test data (5). The 30-s test time is suitable for large-scale epidemiological studies, and is also suitable for self- evaluation of the subject group, whose recorded value is the number of times, which is easier to establish evaluation criteria. According to the literature and the results of the preliminary experiment, in order to ensure the safety of the subjects and the validity of the test results, this study used 30s upper arm dumbbell bending and 30s sitting test for the muscle strength test of the older people aged 70–79 years old, and used 5 times upper arm dumbbell bending and 5 times sitting test for the older people subjects over 80 years old. The test results of this study, the three test results related to muscle strength all confirmed that the muscle strength of the older people over 70 years old showed a gradual decline trend with the increase of age, and the decline was large, and was not affected by gender, except for the female group 70–74 years old and 75–79 years old group, which had no significant decline (*p* > 0.05). There were significant differences among other groups (*p* < 0.05), meanwhile, the results of line chart showed that the indexes of muscle strength decreased the most in 75–79 years old group and 80–84 years old group. This is consistent with the results of previous studies showing that the muscle strength of older people after 80 years old declines and shows an obvious inflection point (25), and this may be related to the ultrastructural changes of muscle cells in the older people around 80 years old. With the increase of age, the number of muscle cells in the older people decreases, some muscle cells become necrotic, and the number of cholinergic neurons decreases resulting in the reduction of motor units, which is the direct cause of the decline in muscle strength and explosive power. At the same time, the muscle strength quality test results of older people over 70 did not show the synergism of upper and lower limb muscle strength. The analysis of the reasons may be related to the different types and amounts of physical activities of the older people, and the local activities lead to the lower and upper limbs failing to achieve the same exercise effect, suggesting that the older people should carry out more systemic exercise to achieve the synergistic development of upper and lower limb muscle strength.

#### 4.3.2 Flexibility quality

It is not appropriate to use the traditional flexibility test method when studying the flexibility of the older people. Due to the degenerative changes in the shape, structure and physiological function of the older people, due to the poor flexibility of the posterior ligaments, tendons and muscles of the hip and knee joints, and most of the older people are fat and have large abdomens, it is very difficult to complete the “sitting forward bend” test commonly used in the flexibility of teenagers. According to the characteristics of the older people, the test method of chair seat forward bending (CSR method) was selected. Houman et al. (26) compared a variety of flexibility test methods for the older people over 70 years old, and concluded that the forward bending of the seat body has the characteristics of simple operation, strong feasibility and good stability. The way of hands hook back is an effective index to evaluate the upper limb flexibility of the older people (27). Therefore, in this study, the upper limb and lower limb flexibility of the older people over 70 years old were, respectively, measured by the back hook of the hands and the forward bend of the seat body in the flexibility test. The results show that the mean flexibility of the older people over 70 years old in this study is at a lower level compared with the fitness health norms of the United States (28), and it increases with age. The upper and lower limb flexibility of the older people at all ages decreased significantly (*p* < 0.05), which is consistent with the results of Chen Xiaoxia et al. (6) 's study on the low flexibility of the urban older people and its rapid decline after the age of 70. From the perspective of gender, females are slightly better than males, which is consistent with the anatomical structure and physiological characteristics of the female body. The rapid decline of flexibility in the older people is closely related to the decline of muscle strength and bone density with age, and it is also prone to fall, fracture and disability. It is suggested that the older people should appropriately increase the range of joint activity when participating in sports activities, combined with strength exercise, in order to improve or maintain the flexibility of the body.

#### 4.3.3 Aerobic endurance

The “gold standard” of endurance quality is maximal oxygen uptake. At present, the endurance quality commonly used in physical fitness testing is achieved through step test (29), step test is an indirect measurement method of maximal oxygen uptake, and 2 min standing in place is considered to be an improved version of step test, and the two have a good correlation. Step test requires testing at a certain rhythm. However, many older people cannot maintain a fixed rhythm, and the result of the 2 min standing in place test is to calculate the number of times completed by the subjects within 2 min, the older people can adjust the speed and rhythm by themselves, and the number of times that do not reach the specified height can be eliminated. During the test, the test staff subjects. Therefore, this study chose 2 min standing in place, which has low requirements for conditions, is easy to operate, implement and record, and is easy to promote and form an exercise pattern, to complete the endurance test of older people over 70 years old. The results showed that the endurance quality of the older people over 70 years old showed a gradual decline trend with the increase of age, and the difference among all age groups was significant (*p* < 0.05). Combined with the results of quiet pulse and vital capacity in this study, the cardiopulmonary function of the older people over 70 years old gradually declined with the increase of age. It is suggested that the older people over 70 years old should plan to complete aerobic capacity exercises in order to maintain endurance quality, protect cardiopulmonary function, and reduce the incidence of cardiovascular diseases.

#### 4.3.4 Balance

Slowing the decline of balance ability is of great significance for the older people to prevent falls and complete daily living behaviors. However, the World Health Organization, the American College of Sports Medicine and domestic physical fitness research fields have not included balance ability in the evaluation of physical fitness or physical fitness. However, Wang Hongyu (12) conducted semi-structured interviews with experts in his research, and the results showed that the experts agreed that balance ability is very important for the older people, and it can be included in the physical fitness test index of the older people over 70 years old. Zhang (30) evaluated the fall risk of the older people and selected three test methods: standing with single foot closed eyes, gait test, and timing test of sitting-standing walking. The results showed that balance ability was highly correlated with fall risk. Standing with single foot closed eyes is a common index of national physical fitness test. However, if the older people over 70 years old are tested with this method, the risk of falling is too high, so it is excluded. The gait test should be carried out in laboratory conditions and is not suitable for outdoor testing. In this study, 2.44 m chair was used to stand up and walk around objects to complete the dynamic balance ability test, which is also an international index Previous studies have found that the balance ability of the older people begins to decline at the age of 60, before which the balance ability of adults is relatively constant and can be maintained at a high level, after the age of 60, the trend of 16% decline every 10 years, and the decline rate is even greater after the age of 80 (7). The results of this study further confirmed this view. The average time for the older people over 70 years old to complete the 2.44 m chair standing up and walking around the object gradually increased with the increase of age, and the differences among all groups were statistically significant (*p* < 0.05), and the average time for each group to complete the test activities was higher than that for the older people of the same age in the United States (28). The results showed that the homeostasis ability of older people over 70 years old was worse than that of older people in the same age group in the United States, and it showed a decreasing trend with the increase of age, and male was better than female. It is suggested that we should pay attention to the evaluation and training of balance function in the older people over 70 years old, delay the decline of balance ability with age, control the risk factors of falls, prevent the occurrence of falls, and improve the quality of life of the older people.

## 5 Conclusion

In this study, the behavioral ability and physical status of the older people in all age groups were effectively understood through physical testing of the older people over 70 years old, and the aging trend of each single index of the older people over 70 years old was analyzed. The aging characteristics were mainly shown as follows:

1. Vital capacity, flexibility, muscle strength, aerobic endurance and balance ability all decline significantly with age, and 80 years old is the inflection point of rapid decline in various indicators.
2. Blood pressure, quiet pulse, BMI, waist-to-hip ratio and waist-to-height ratio did not change with age.
3. Blood pressure, BMI, waist-to-hip ratio, waist-to-height ratio and other indicators test results reflect that the older people over 70 years old is a high-risk group of metabolic diseases, cardiovascular and cerebrovascular diseases.

## 6 Contribution and limitation

This study conducted physical fitness test on the older people over 70 years old in 7 geographical regions of China, which is the first nationwide physical fitness test for the older people, which is an extension and expansion of the national physical fitness monitoring system, and also shows that the test indicators involved in the “Health fitness scale” are simple and feasible. And the study added a series of test data over 70 years old, which is the basis for scientific and reasonable formulation of physical fitness evaluation standards for the older people, and is of great significance for improving the national physical fitness database and grasping the dynamic changes of national physical health status, and providing data support for scientific guidance of physical exercise for the older people.

At present, there is still a lack of national physical evaluation standards for the older people over 70 years old, so they cannot be comprehensively scored. In the study, the older people over 70 years old were divided into groups only in terms of age and gender, and other factors related to physical fitness (regional, urban and rural, etc.) may not be considered. Whether more medical indicators should be added to the physical health evaluation of the older people needs to be further explored and studied.

## Data availability statement

The raw data supporting the conclusions of this article will be made available by the authors, without undue reservation.

## Ethics statement

Ethical approval was not required for the studies involving humans because ethical approval was not required for the study involving human samples in accordance with the local legislation and institutional requirements. The studies were conducted in accordance with the local legislation and institutional requirements. The participants provided their written informed consent to participate in this study.

## Author contributions

J-BS: Conceptualization, Funding acquisition, Investigation, Methodology, Project administration, Validation, Writing – review & editing. Y-YM: Data curation, Formal analysis, Methodology, Resources, Supervision, Writing – original draft. QN: Software, Visualization, Writing – original draft.
